# Assessment of genetic diversity and population structure in wild *Ziziphus* species from northwest India using SSR marker technique

**DOI:** 10.1186/s43141-022-00458-6

**Published:** 2023-01-13

**Authors:** Amit Sareen, Vikas Sharma, Raghbir Chand Gupta

**Affiliations:** 1 Department of Botany, Punjabi University Patiala, Patiala, Punjab India; 2 Department of Agriculture, Sant Baba Bhag Singh University Khiala, Jalandhar, 144030 India

**Keywords:** *Ziziphus mauritiana*, *Z. nummularia*, Simple sequence repeats, Genetic diversity, Population structure, Polymorphism information content

## Abstract

**Background:**

*Ziziphus* species particularly *Ziziphus mauritiana* and *Ziziphus nummularia* constitute an important part of genetic resources in India. They contribute economically as a fruit crop with lots of morphological and pomological variability. In current study, 48 accessions belonging to two wild *Ziziphus* species, i.e., *Z. mauritiana* and *Z. nummularia*, were characterized using SSR markers. In addition, external features were also examined using stereomicroscope.

**Results:**

Present investigation was done to explore the genetic structure of North Indian jujube. In total, 23 SSR markers detected 57 SSR alleles with an average of 2.47 alleles. Highest number of alleles (4) were detected by three primers, namely BFU1178, BFU479, and ZCMS14, while lowest number of alleles (2) were detected by fifteen primers. Highest Polymorphism Information Content (PIC) was 0.500 and shown by two primers, namely BFU528 and BFU1248, while lowest PIC (0.041) was observed in primers BFU286 with mean value of 0.443. Similarly, highest value of marker index (MI) was detected by primer BFU1178 i.e. 1.969, and lowest value of marker index was observed in primer BFU286 i.e. 0.021. Dendrogram generated using SSR markers data and principal component analysis showed two major groups of the analyzed germplasm with intermixing. STRUCTURE analysis also clustered all the accessions into two groups. We did not found correlation between geographic and genetic distances.

**Conclusions:**

The preliminary results suggest that there is high level of gene pool mixing in these species which can be attributed to their cross-pollination habit. However, more such studies with large numbers of samples are required in future to gain concrete insights of the genetic structure in these species.

**Supplementary Information:**

The online version contains supplementary material available at 10.1186/s43141-022-00458-6.

## Background

**Fig. 1:**
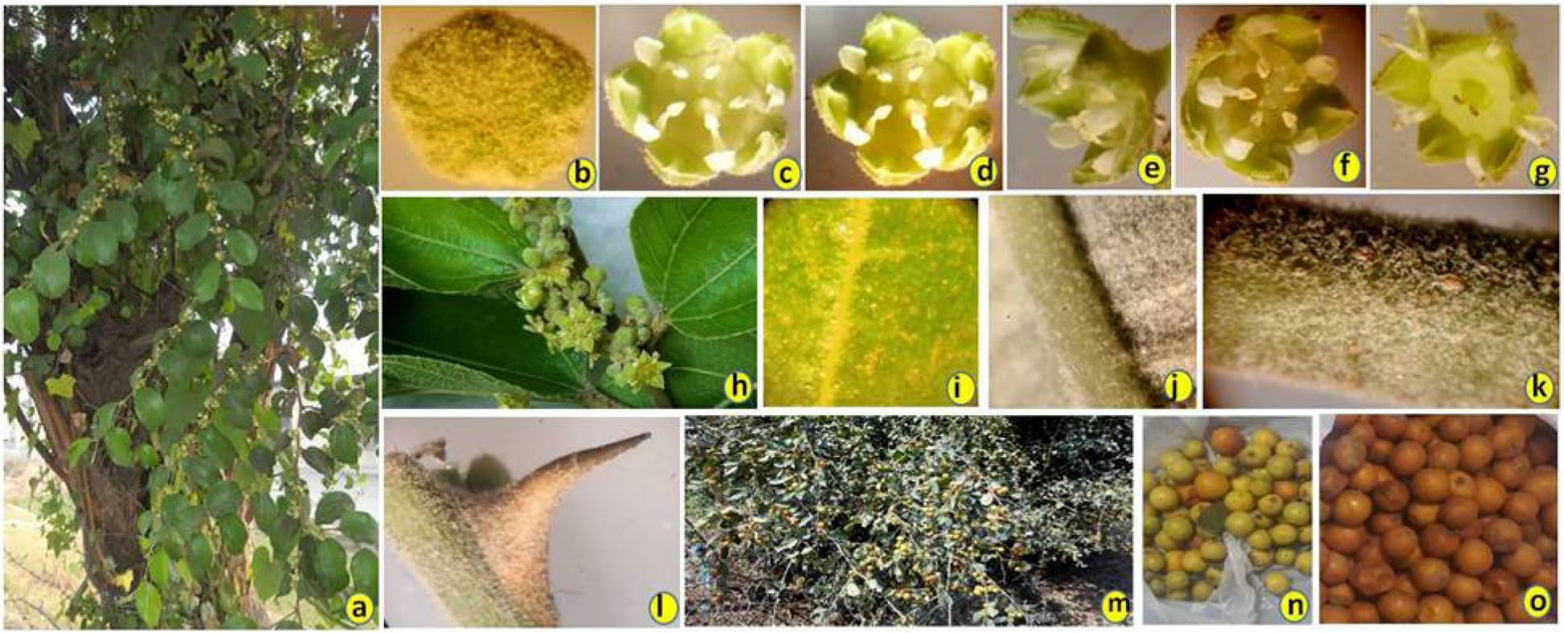
Morphological features of Z. *mauritiana* and Z. *nummularia*. **a**) Mature plant of *Z. mauritiana*. **b**) Flower bud of Z. *mauritiana* photographed with stereomicroscope. **c**–**g**) Stereomicroscopic photographs of flower showing anthers and stigma. **h**) A flowering branch. **i**) Abaxial leaf surface under stereomicroscope. **j**) Adaxial leaf surface under stereomicroscope. **k**) Stem surface under stereomicroscope. **l**) Prickle under stereomicroscope. **m**) *Z. nummularia* plant-bearing fruits. **n**–**o**) Unripend and ripened fruits of *Z. nummularia*

*Ziziphus mauritiana* and *Ziziphus nummularia* both jujube species are commonly known as ber (Indian jujube) in India. These are subtropical to tropical plant species, and fruits of these species are liked and eaten by large population of India. *Z. mauritiana* is a cultivated species, while *Z. nummularia* is a wild species, and both of these vary in their habits. Generally, both the species have prickly stems and branches with ovate leaves and minute flowers. The pollination is strictly cross-pollination as it also shows the protandry habit. However, plants of *Z. mauritiana* are larger in height having larger leaves, stems, branches and fruits, while plants of *Z. nummularia* show shrub-type habit with smaller leaves, profuse branching and small fruits [46, 58, 64]. Some morphological features of both of these species are shown in Fig. 1. Both of these species play important role in economy as well as in ecology. Fruits of these species contain many important constituents such as vitamins, alkaloid, and other secondary metabolites which exhibit many health benefits [34, 35, 37, 40]. However, with the introduction of new hybrid cultivars in the market, the wild genetic resources are being neglected which is the matter of concern. We do not know the type and diversity of wild genetic resources we are having at present. However, this type of information is highly required for conservation of important genetic resources. The effective conservation, management, and efficient utilization of plant genetic resources can be done if we have explored the basic knowledge about essential biological phenomena in plants and characterize them timely. An adequate knowledge regarding how to best utilize the existing genetic diversity in plant population is of fundamental interest for the efficient management of plant resources [28, 62]. Characterization of genetic resources includes many ways such as morphological traits, chemical compounds identification, genetic traits and cytological studies. To include all these techniques or methods in a single study is somewhat tedious and cumbersome and needs expertise at all these levels. The most common characterization method is morphological characterization; however, it suffers from few limitations such as varying of phenotypic traits with varying environments [50, 61] and sometimes results in wrong interpretations and conclusions. Therefore, a good alternative to this is the characterization at genetic level using DNA markers or genetic markers [39, 54, 66, 67]. These marker techniques require expertise and few advanced instrumentation, but results are reliable and free from any limitations. Genetic markers are determined by allelic forms of genes or genetic loci or polymorphic fragments of DNA and can be stably transmitted from one generation to another. Therefore, these markers can be used as experimental tags to keep track of an individual, a tissue, a cell, a nucleus, a chromosome, or a gene. Genetic markers are broadly categorized into two main categories, i.e., classical markers and DNA markers [69]. Classical markers include morphological markers, cytological markers, and biochemical markers. DNA markers are the fragments of DNA revealing polymorphism between different genotypes or individuals or alleles of a gene. The polymorphism shown by marker fragments may arise due to alteration of nucleotide or mutation in the genomic loci [20]. These fragments are associated with a defined location within the genome and may be detected by means of different molecular marker techniques such as restriction fragment length polymorphism (RFLP), randomly amplified polymorphic DNA (RAPD), amplified fragment length polymorphism (AFLP) and single-nucleotide polymorphism (SNP) [12]. Molecular makers have been established as powerful tools in the analysis and assessment of genetic variation as well as in establishing genetic relationships within and among species [9, 29, 43, 44, 50, 53, 55]. There are great advantages of molecular markers as compared to traditional morphological markers. Molecular markers exhibit high polymorphism, reproducibility, even distribution across the whole genome, and selectively neutral behavior to environmental conditions. Therefore, it is used in many different areas such as genetic mapping, diversity analysis, parentage analysis, pedigree analysis, gene identifications, fidelity checking of tissue culture raised plants, and many more areas in breeding of crops and population genetic studies [4, 7, 8, 21, 25, 27, 33, 48, 49, 51]. However, among different marker systems, simple sequence repeat (SSR) markers have become the markers of choice due to their easy availability, codominant nature, and easy detection and cross-transferring nature across species and genera [3, 24, 52]. In Indian jujube species, few studies related to morpholoical and molecular markers have been reported [2, 6, 10, 13, 19, 22, 30, 31, 36, 56–60, 70]. However, most studies were conducted using less number of samples and dominant markers such as RAPD and AFLP. Moreover, Indian jujube germplasm has been explored less and requires more molecular works. Therefore, in present study, we have utilized the SSR markers in *Ziziphus* species with specific objectives to characterize the wild and cultivated genetic resources of *Ziziphus* in north western Indian states and to establish genetic relationships among the analyzed accessions of both the species, i.e., *Z. mauritiana* and *Z. nummularia*.

## Materials and methods

### Plant materials and DNA extraction

In the present study, 48 *Ziziphus* accessions i.e. 20 accessions of *Z.mauritiana* and 28 accessions of *Z. nummularia*, were collected from different geographical locations of northwest Indian states (Punjab, Rajasthan, Haryana, and Himachal Pradesh) and were analyzed using SSR markers. Of these, thirteen samples were from Punjab, twelve from Rajasthan, twelve from Haryana, and eleven from Himachal Pradesh. A detailed description with locations of all the accessions and their altitude range is given in Table 1. Young and fresh leaf samples belonging to these plants were collected. Leaves were properly observed, and effort was made to check that samples were free of disease or any damage. Samples were put in an airtight (sterilized) plastic bag containing silica gel to prevent the moisture and subsequent degradation. DNA was extracted using CTAB method [14] and liquid nitrogen.

**Table 1:** Details of forty-eight accessions of two species analyzed in the present study

S. no.	Species	Location	Code	Elevation	Geographical coordinates
1	*Ziziphus mauritiana*	Patiala	Pb-1	250 m	30°20′24″ N, 76°22′ 47″ E
2	*Z. mauritiana*	Patiala Baradari	Pb-3	250 m	30°20′23″ N, 76°22′ 46″ E
3	*Z. mauritiana*	Sanaur	Pb-4	253 m	30°30′23″ N, 76°46′ 15″ E
4	*Z. mauritiana*	Thapar, Patiala, University	Pb-9	252 m	30°21′10″ N, 76°22′ 16″ E
5	*Ziziphus nummularia*	Ropar	Pb-10	260 m	30.97° N, 76.53° E
6	*Z. nummularia*	Bassi Pathana	Pb-11	247 m	30.68° N, 76.40° E
7	*Z. nummularia*	Patran	Pb-18	240 m	29.9593° N, 76.0566° E
8	*Z. mauritiana*	Moonak 1	Pb-20	241 m	29.8253° N, 75.8912° E
9	*Z. mauritiana*	Samana	Pb-22	240 m	30.1554° N, 76.1958° E
10	*Z. nummularia*	Punjabi Uni	Pb-23	252 m	30.36° N, 76.45° E
11	*Z. mauritiana*	Moonak 2	Pb-25	241 m	29.825o° N, 75.8910° E
12	*Z. mauritiana*	Devigarh 1	Pb-31	256 m	30°26'13″ N, 76°29′ 16″ E
13	*Z. nummularia*	Devigarh 2	Pb-33	256 m	30°24'13″ N, 76°28′ 19″ E
14	*Z. nummularia*	Rajasthan, Churu	Rj-35	292 m	28.26° N, 74.89° E
15	*Z. nummularia*	Rajasthan, Churu	Rj-36	312 m	28.30° N, 74.95° E
16	*Z. nummularia*	Rajgarh	Rj-37	479 m	28°37′50.66″ N, 75°20′ 50.24″ E
17	*Z. nummularia*	Rajgarh	Rj-38	479 m	28°39′50.69″ N, 75°22′ 50.26″ E
18	*Z. nummularia*	Gulpura	Rj-39	392 m	29°44°N, 69.60°E
19	*Z. mauritiana*	Hanumangarh	Rj-41	177 m	29°31′ 0″ N, 74°15′ 0″ E
20	*Z. nummularia*	Hanumangarh	Rj-42	177 m	29°35′ 0″ N, 74°19′ 0″ E
21	*Z. mauritiana*	Ganganagar	Rj-43	178 m	29°54′13.8204″ N, 73°52′ 37.8840″ E
22	*Z. nummularia*	Jaipur	Rj-44	431 m	26°55′10.6″ N 75°47.269′ E
23	*Z. nummularia*	Alwar	Rj-45	268 m	27°33′ 39.3552″ N, 76°37′ 30.0540″ E
24	*Z. nummularia*	Ganganagar	Rj-46	178 m	29°52′10.8200″ N, 73°51′ 35.8832″ E
25	*Z. mauritiana*	Hanumangarh	Rj-47	177 m	29°30′ 0″ N, 74°14′ 0″ E
26	*Z. mauritiana*	Haryana, Hisar	Hr-50	215 m	29.09° N, 75.43° E
27	*Z. nummularia*	Haryana, Hisar	Hr-51	215 m	29.11° N, 75.44° E
28	*Z. mauritiana*	Barwala	Hr-52	214 m	29.06° N, 75.39° E
29	*Z. nummularia*	Barwala	Hr-53	214 m	29.09° N, 75.43° E
30	*Z. mauritiana*	Chutala	Hr-54	215 m	29.7808° N, 74.5221°E
31	*Z. mauritiana*	Tohana	Hr-55	229 m	29.7163° N, 75.9057° E
32	*Z. nummularia*	Tohana	Hr-56	229 m	29.7157° N, 75.9048° E
33	*Z. nummularia*	Jind	Hr-57	227 m	29.32° N, 76.29° E
34	*Z. nummularia*	Yamunanagar	Hr-58	255 m	30.08° N, 77.22° E
35	*Z. nummularia*	Yamunanagar	Hr-59	255 m	30.10° N, 77.28° E
36	*Z. mauritiana*	Cheeka	Hr-63	252 m	30.049° N, 76.342° E
37	*Z. mauritiana*	Kaithal	Hr-64	252 m	29.8043° N, 76.4039° E
38	*Z. nummularia*	Himachal Damooni	Him-1	730 m	31°55′26.81″ N, 76°47′ 3.77″ E
39	*Z. nummularia*	Dehradun	UK-48	435 m	30°18′59.3856″ N, 78°1′ 55.8768″ E
40	*Z. nummularia*	Hamirpur	Him-3	738 m	31°39′26.79″ N, 76°29′ 3.73″ E
41	*Z. nummularia*	Awahldevi	Him-4	700 m	31°38′26.77″ N, 76°30′ 3.74″ E
42	*Z. nummularia*	Hamirpur	Him-5	738 m	31°41′26.81″ N, 76°31′ 3.77″ E
43	*Z. mauritiana*	Hamirpur	Him-6	738 m	31°40′26.88″ N, 76°30′ 3.75″ E
44	*Z. mauritiana*	Una	Him-49	389 m	29°35′0″ N,74°19′ 0″ E
45	*Z. nummularia*	Kangra	Him-8	733 m	32°5′59.26″ N, 76°14′ 8.75″ E
46	*Z. nummularia*	Kangra	Him-60	733 m	32°5′59.29″ N, 76°16′ 8.77″ E
47	*Z. nummularia*	Paonta Sahib	Him-65	389 m	30°42°N, 77.57°E
48	*Z. mauritiana*	Paonta Sahib	Him-11	389 m	30°44°N, 77.60°E

### Simple sequence repeat reactions

Thirty-one SSR primers which were developed by Wang et al. [65] were analyzed for polymorphism on a forty-eight selected DNA samples from *Ziziphus* species from various locations of northwest India (Table 1). Out of these, twenty-three primers were concluded as good reliable with unambiguous amplification and were further used for genotyping. SSR amplifications were carried in a 10 μl volume which was constituted using 4.8 μl of sterilized distilled water, 2.0 μl genomic DNA (13 ng/μl), 0.5 μl of forward and 0.5 μl of reverse primer (5 μM), 0.5 μl MgCl_2_ (25 mM), 1.0 μl 10 × PCR buffer (10 mM Tris-Hcl, 50 mM Kcl, pH 8.3), 0.5 μl dNTP mix (0.2 mM each of dATP, dGTP, dCTP, and dTTP), and 0.2 μl *Taq* polymerase (5 U/μl). The PCR conditions were as follows: 1 cycle of 5 min at 94 °C, 35 cycles of 1 min at 94 °C, 1 min at respective annealing temperature for each primer as shown in Table 2, 1 min at 72 °C, and final extension for 7 min at 72 °C. Amplification products were separated on 3% agarose gel in 1 × TBE buffer, and size of each fragment was estimated 50 bp DNA ladder (MBI Fermentas, Lithuania). Fragments were visualized by using ethidium bromide, and permanent photographs of gels were taken in gel documentation system (Bio-Rad laboratories-segrate, Milan, Italy).

### Data analysis

Only unambiguously amplified alleles were scored manually and converted into binary data, i.e., 1 for the presence of band and 0 for the absence of band. Polymorphism Information Content (PIC) values were calculated using the formula given by Botstein et al. [5, 26]. Distance-based cluster analysis was performed by generating dendrogram based on Jaccard similarity coefficient and UPGMA method using DARwin [41]. The population genetic structure was elucidated using Bayesian model-based clustering method implemented in the software STRUCTURE, version: 2.3.3 [17, 42]. Ancestry model with admixture and correlated allele frequency model was set to get the estimates of posterior probability of data. Ten independent runs were given setting the value of K from 1 to 10 with 3 iterations for each value of K. Length of burn-in period was set at 100,000, and number of Markov chain Monte Carlo (MCMC) repeats after burn-in was set at 100,000. Evanno’s method [16]-based program STRUCTURE HARVESTER developed by Earl and Vonholdt [15] was utilized to find the value of estimated Ln probability of data LnP(K) and to get the best fit value of K for the data. STRUCTURE was run for all the analyzed accessions of the two species. Analysis of molecular variance (AMOVA) and Mantel test were performed using GenAlEx 6.5 version.

## Results

### SSR polymorphism and population structure

In the present study, twenty-three SSR primers were utilized which amplified unambiguously and produced reliable alleles. In total, 23 SSR primers amplified 57 alleles with an average of 2.47 alleles per primer. Size range of alleles varied from 80 to 500 bp. Minimum 2 alleles were amplified by fifteen primer pairs, while highest numbers of alleles were 4 and amplified by three primers as shown in Table 2. Although alleles amplified on average were not high, but considerable polymorphism was detected with these primers. Highest Polymorphism Information Content (PIC) was 0.500 and shown by two primers, namely BFU528 and BFU1248, while lowest PIC (0.021) was observed in primer BFU286 with mean value of 0.443. Similarly, highest value of marker index was detected by primer BFU1178 i.e. 1.969, and lowest value of marker index was observed in primer BFU286 i.e. 0.021. These values will be helpful in identifying suitable primers for future use in different genetic studies. STRUCTURE analysis of these two species showed two populations and the log likelihood reached a clear maximum value at *K* = 2 (Fig. 5a). Population structure showed that two different gene pools were contributing in the genetic makeup of analyzed accessions (Fig. 5b).

**Table 2:** SSR primers used in the present study with the details of alleles amplified and diversity indices

Primer name	Primer sequence	Repeat motif	Ta (°C)	Alleles	Size range (bp)	Ho	He	PIC	MI
BFU0263	F-GGTTTTTGTGGGTATGGAGGT R-AGGAAAACAAAGGGATGGAGA	(CT)11	50	3	120–280	0.583	0.434	0.49	1.49
BFU0286	F-GATTGTTGCTGGTTTCCATGT R-CTGGACTCTCCGATGCAGTAG	(AG)10	51	2	160–170	1.000	0.505	0.02	0.04
BFU0377	F-CCAGCTGGTATCCAATTGCT R-ACGACGATGCCATGAAAGAT	(CT)10	50	2	300–330	0.708	0.544	0.45	0.90
BFU0473	F-GTCCTGATGTGGAGTGCATTT R-TCTACAAGGACGAATCGTTGC	(AG)9	52	2	190–200	0.042	0.538	0.33	0.66
BFU0581	F-TGAGAAGGTTGAAGATGCTCTC R-CCTGACATCCATTTGAAGGAA	(CA)7	50	3	80–150	0.708	0.559	0.44	1.34
BFU1157	F-TCCCTAAATTACCCTTCCCAAT R-AAAGCGACAGCGAAAACTGT	(GA)9	50	2	120–130	0.021	0.595	0.35	0.70
BFU1205	F-TGTTGCTGGTTCAATTCCAG R-CTTATGGCTTTTTCATTTTGTGA	(CA)8	48	2	80–100	0.188	0.651	0.48	0.97
BFU1409	F-CAAATGATGGATCGAGCAAA R-AATGGAGGACAAACCGTCAC	(CA)6	48	3	80–100	0.396	0.644	0.49	1.49
BFU1178	F-CCTTGGTGGATTTTGGTTTG R-TATACTTTGGCAGCGGTGTG	(TG)9	50	4	100–250	0.625	0.619	0.49	1.96
BFU0308	F-TTTCCACCCCAAAATACCAA R-AGACGCTGGATGAGGATGAT	(TC)11	49	3	150–450	0.104	0.293	0.46	1.39
BFU0083	F-TTTTCCAACCCTCCCTCCA R-CCTCATAACTGCGACGGCTT	(CT)13	51	2	90–100	0.083	0.557	0.36	0.72
BFU0467	F-CCGGACCGAGTGGAGTTATTA R-AGAATATGGCATCAACCTATACCA	(TC)9	52	2	100–130	0.250	0.649	0.48	0.97
BFU0528	F-TTTGTGAGGTATAATGGCTTTCA R-GCCTCTGTTGAAGCAAGGAA	(TC)8	50	2	100–150	0.063	0.442	0.50	1.00
BFU1248	F-TCCCACCACTTTCCTCTCAT R-TTTTTCAAGACCTCCACGATG	(ATTA)4	50	2	100–300	0.000	0.434	0.50	1.00
BFU1279	F-TTTTTCAAGACCTCCACGATG R-TCCCACCACTTTCCTCTCAT	(TTAA)4	50	2	150–200	0.271	0.511	0.48	0.97
BFU0249	F-AATGGGTCCACGTAGACAGG R-GCCCTGAGGTTGGACATAGA	(GT)12	54	2	200–250	0.083	0.483	0.49	0.99
BFU0561	F-CCAGATGTGTCTCGATGCTT R-CCAGATGTGTCTCGATGCTT	(CT)7	52	2	350–500	0.125	0.482	0.49	0.99
BFU0574	F-GAAGGTTGAAGATGCTCTCTCTC R-CCTGACATCCATTTGAAGGAA	(CA)7	51	3	100–250	0.271	0.639	0.44	1.33
BFU0479	F-GAAAACCATTGTTGGAGACCA R-TGAACCAAGCAACAAAAATCA	(TC)9	47	4	250–450	0.563	0.776	0.48	1.92
ZCMS14	F-GAAGCTCCAATAACACGTTACC R-ACAATTCCCCAAATCTAAACTG	(AG)8	49	4	300–500	0.854	0.719	0.45	1.80
ZCMS1	F-CTCATCTTCTAAAACCAAAAACC R-CTCTGTCAACATATCTGGCTTG	(AG)10	50	2	100–200	0.313	0.641	0.49	0.98
ZCMS2	F-CTTCTAAAACCAAAAACCCTTC R-CTCTGTCAACATATCTGGCTTG	(GA)12	49	2	100–150	0.479	0.653	0.47	0.95
ZCMS11	F-CAACTCTGCATCAAATCCATC R-TGACTGTTCCGATAATTTCAAC	(GA)8	49	2	300–400	0.063	0.460	0.47	0.94
Mean				2.47		0.063	0.442	0.44	1.11

*Ta* annealing temperature, *Ho* observed heterzygosity, *He* expected heterozygosity, *PIC* polymorphism information content, *MI* marker index

**Fig. 2:**
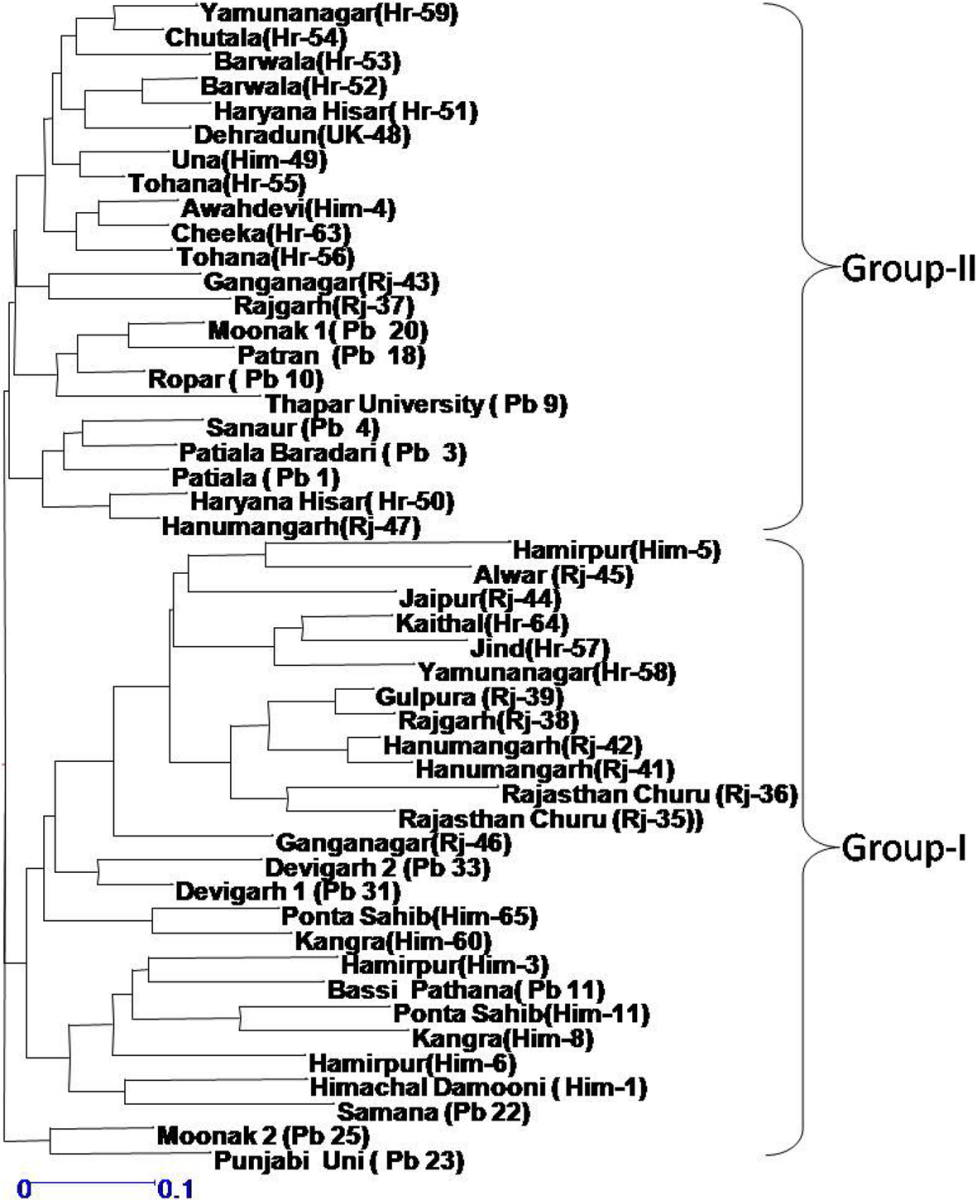
Dendrogram of 48 *Ziziphus* accessions based on 23 SSR markers data showing clustering of all accessions into two major groups

**Fig. 3:**
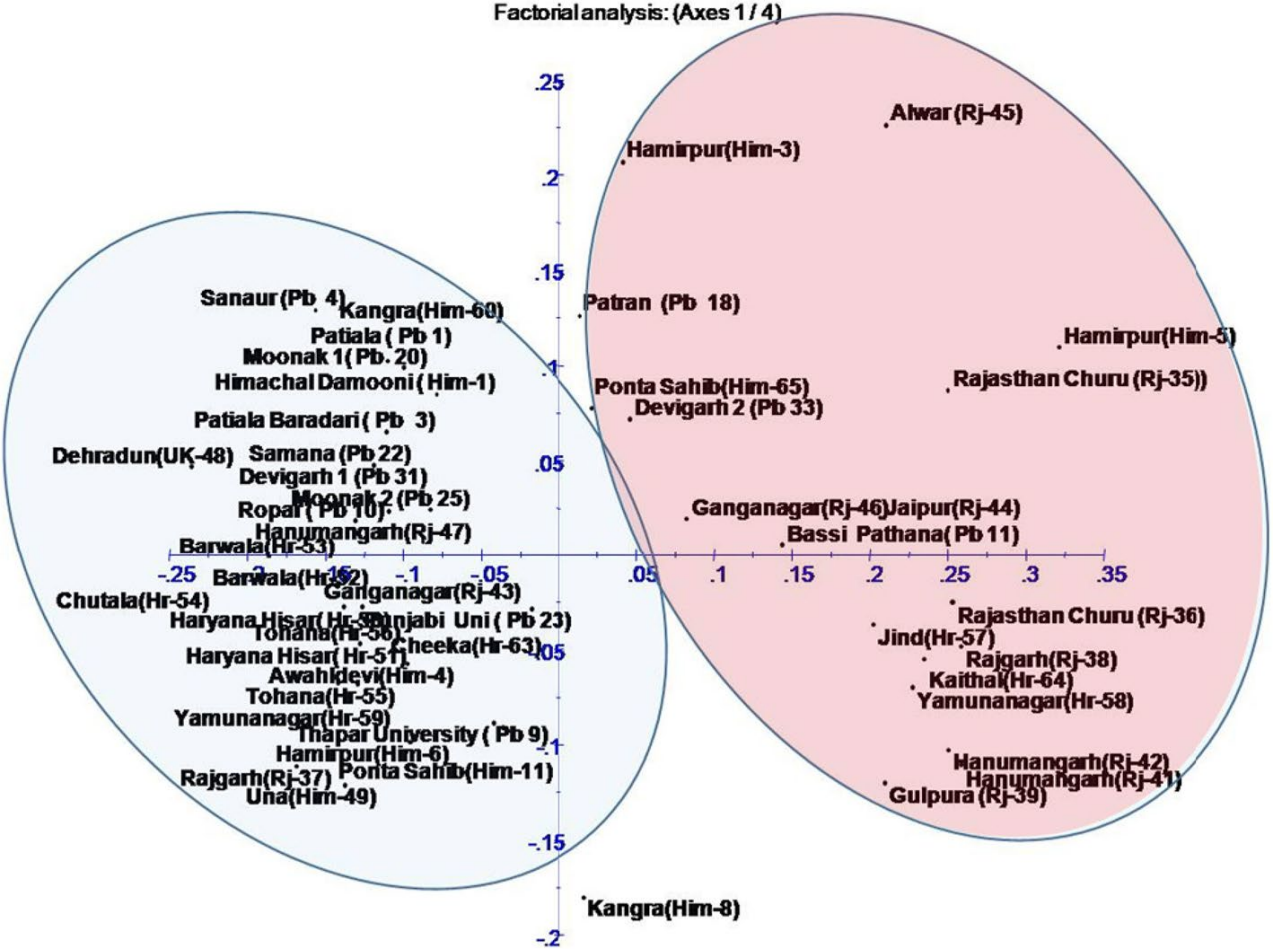
Principal coordinates analysis based on Eigenvalues calculated from 23 SSR markers. The 48 accessions were assigned into two groups

**Fig. 4:**
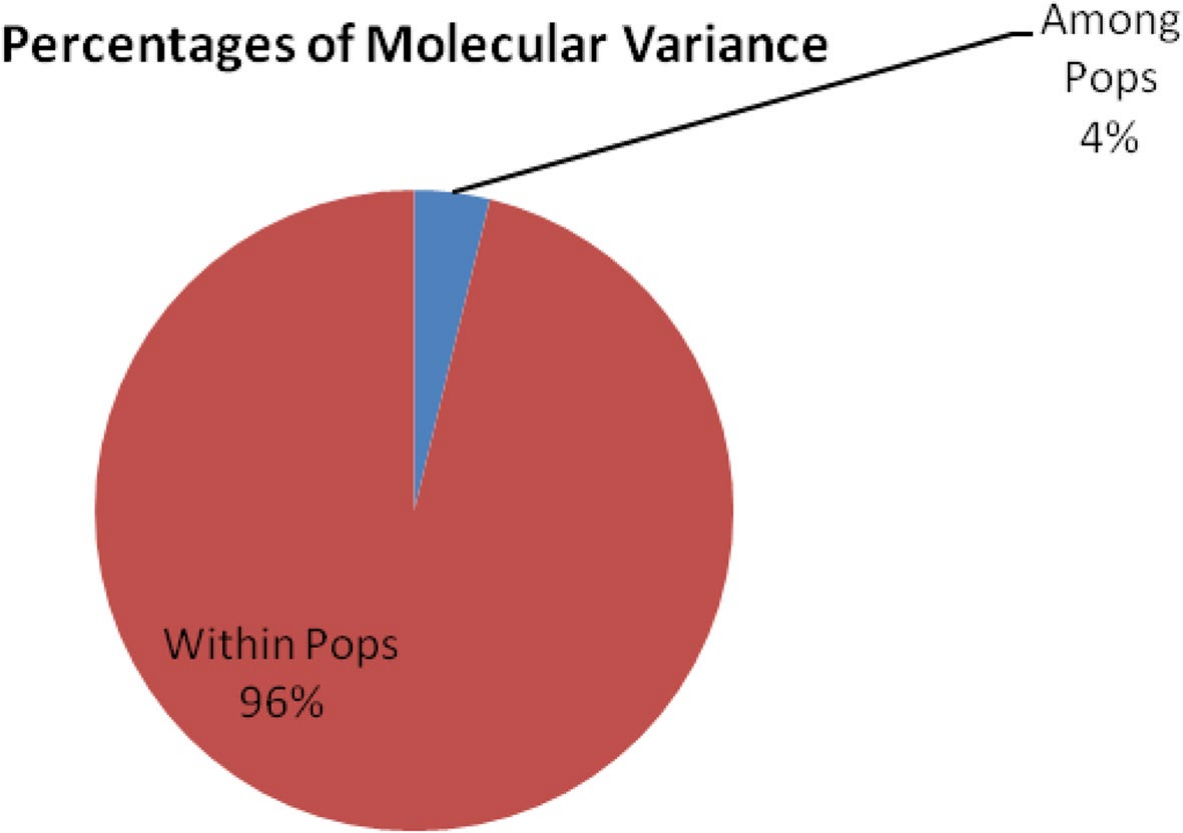
AMOVA showing genetic variance within and among populations

When cluster analysis of the studied species was done using dendrogram and principal component analysis (PCA), two major groups (Figs. 2 and 3) were observed. Each of this group was formed of accessions from each of the studied species. Group 1 consisted of twenty-four accessions from different geographical regions and mixed accessions of both the species under investigation. The subgroups of group 1 consisted largely on the basis of geographical locations rather than species basis. Group 2 contained accessions from different states like Himachal Pradesh, Rajasthan, Haryana, and Uttarakhand, but majority of accessions from Haryana grouped in this cluster, and out of twelve, the nine accessions from Haryana were included in this group. Two accessions, namely, Moonak 2 (Pb25) and Punjabi Uni (Pb23), remained as outlier and grouped outside the two major groups. AMOVA showed 96% variance within populations and 4% variance among population (Table 3 and Fig. 4). Mantel test showed nonsignificant correlation between geographic and genetic distance (Supplementary Fig. 1).

**Table 3:** AMOVA showing genetic variance within and among populations

Summary AMOVA table					
Source	df	SS	MS	Est. var.	%
**Among pops**	1	19.063	19.063	0.391	4%
**Within pops**	46	456.729	9.929	9.929	96%
**Total**	47	475.792		10.320	100%

## Discussion

### SSR diversity and structure

Genetic diversity and population structure of *Ziziphus* germplasm from India is needed for its improvement in future and for the conservation of diverse and promising accessions. Genetic diversity gives the estimates of DNA polymorphism of the analyzed germplasm, and this polymorphism can be used in future for improving and manipulating the germplasm for various purposes. In the genus *Ziziphus*, the characterization of the cultivars had been largely based on morphological characters and their uses [32]. However, molecular marker studies have also been initiated at by different research groups at various places. The molecular studies in the 5 ber cultivars (*Z. mauritiana*) of Saudi Arabia were done using ISSR markers and it has been observed that a cultivar called Um-sulaem was paraphyletic to the other four [38] accessions analyzed. In *Z. mauritiana*, some other workers also conducted research using RAPD and ISSR [13, 47]. Furthermore, the two varieties of Indian jujube were also found genetically similar using RAPD markers [63]. The similar study was conducted in the same species using nr DNA and RAPD primers, and intraspecific variations were reported with about 85% polymorphism to separate delineate of the populations into 4 clusters [59]. Most recently, there is a maiden report of using SSR markers in *Z. jujuba* from China, and reported high genetic diversity (98.2%) in corresponding 3 clusters was observed using 31 primer pairs [65]. The present study differs from the previous as the germplasm collected is from diverse geographical locations and inclusion of two species.

**Fig. 5:**
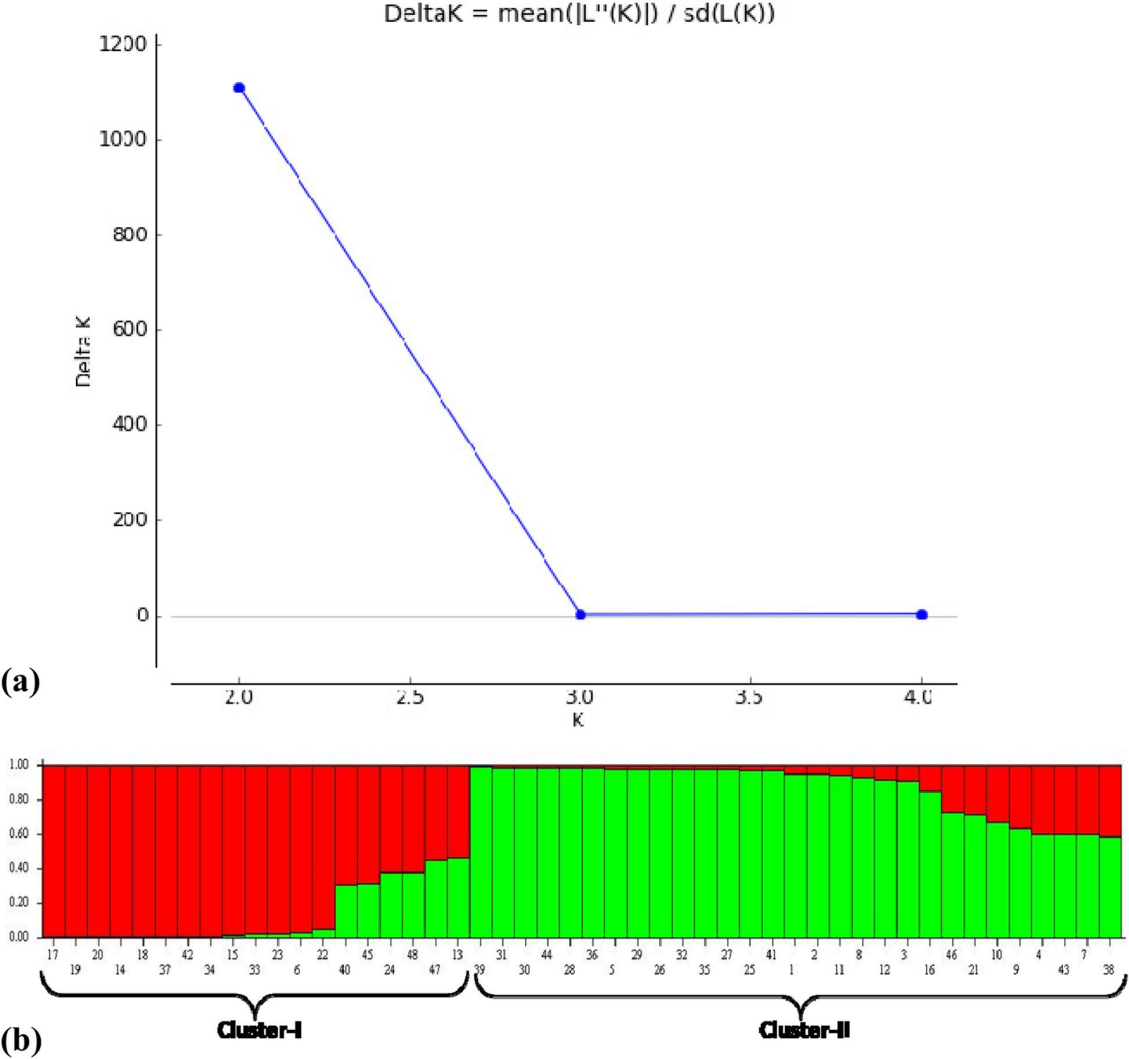
**a** Graph showing the best value of k at 2. **b** Bar plot of 48 accessions of *Ziziphus* showing two clusters by STRUCTURE. These clusters represent two populations with admixed individuals

To investigate the genetic relationship between the domesticated and wild jujube populations, chloroplast microsatellite markers (cpSSR) were developed by Huang et al. [23]. Using these cpSSR markers, the number of alleles per locus was found between two and four which is exactly like the alleles obtained in present study. Furthermore, the values of diversity indices were almost similar to the present study. Chaogun [68] used 24 SSR markers to explore the genetic diversity, genetic structure, and core collection of *Ziziphus jujuba*. STRUCTURE analysis and multivariate analyses (cluster and PcoA) were also done for the grouping of jujube accessions. Fu et al. [18] used SSR markers in Chinese jujube (*Ziziphus jujuba* Mill.) for population genetics, and the average number of alleles per locus was found 12.8 which was much greater than the number of allele obtained in present study. Using 11 ISSR primers to access genetic diversity within and among 34 accessions of *Z. spina-christi* collected from different regions of Saudi Arabia, Saleh Alans et al. (2016) obtained 109 scorable loci, of which 93.4% were found to be polymorphic. The size of amplified bands ranged between 250 and 3000 bp. Significant variability was observed in 10 selected *Z. nummularia* accessions based on various quantitative and qualitative characteristics of leaves, fruits, and seeds. Akhtar et al. [1] used 11 ISSR primers which showed 86.58% polymorphism. A pairwise similarity coefficient among all the 10 accessions were found to ranged from 0.45 to 0.77. Singh et al. [57] investigated genetic diversity among 47 cultivated accessions of *Z. mauritiana* and one wild accession of *Z. nummularia* using 18 ISSR markers; a total 167 products were detected, of which 89.96% were reported polymorphic. Cluster analysis based on UPGMA method and Bootstrap analysis separated all the 48 *Ziziphus* genotypes in 4 distinct clusters, and they were found to be divergent on the bases of Jaccard coefficient. Similar finding was observed in present study as dendrogram is showing divergent clustering as well as mixing of accessions. Saha et al. [45] studied genetic relationship among 26 fruit cultivars of *Z. mauritiana* which included 6 accessions of wild-type *Z. mauritiana*, two accessions of *Z. nummularia*, and 1 accession of *Z. xylopyrus*. The PIC values range between 0.23 to 0.46 and 0.11 to 0.36, respectively, for the RAPD and ISSR primers which are somewhat lower values as observed in present investigation using SSR markers. Thirty-eight microsatellites were isolated from *Z. mauritiana* to evaluate genetic diversity by Chiou et al. [11]. The PIC values ranged from 0.248 to 0.889. The number of alleles per locus ranged from 2 to 13 which were higher as compared to present study. However, most of the research work has been done in China, and Indian germplasm also requires similar works for the proper characterization, utilization, and conservation. Cluster analysis using dendrogram, principal component analysis (PCA), and STRUCTURE of the studied species was done; all of the methods showed two major groups (Figs. 2, 3 and 5). Each of this group was formed of mixed accessions from each of the studied species. Although majority of grouping was according to geographical locations, some exceptional mixing events cannot be neglected, and insights into these events are needed to make the things more clear. As both the species are cross-pollinated and at many sites both species were reported to occur in vicinity to each other, the cross-pollination may be a regular process between these two species. This mixing is also supported by Mantel test that showed nonsignificant correlation between geographic and genetic distances (Supplementary Fig. 1). On the other hand, pollens can be driven by wind to distant locations; these phenomena can be the reasons behind germplasm exchange and mixing. Furthermore, AMOVA indicate that larger portion of variance is within populations rather than among populations.

## Conclusion

In the present study, SSR markers showed high genetic diversity and mixing of gene pools in studied accessions of both jujube species. Results showed that there are two genetic stocks contributing in analyzed accessions. We found no specific correlation between different accessions of same species on the basis of geographical locations. The results of this research work can be useful in future research works in *Ziziphus* species to understand the spread of species and sharing of genomes between wild and cultivated germplasm. Furthermore, identification of diverse accessions based on minute morphological differences as well as at DNA level can be done for conservation and for initiating new breeding programs in *Ziziphus* species.

## Supplementary Information

**Additional file 1: Supplementary Fig. 1** Showing correlation between Genetic and geographic distances of 48 samples used in present study.

**Additional file 2: Supplementary Table 1** Axis wise Eigen values.

## Data Availability

Data analysis details were available from corresponding author on request.
