# Supplementary material for: Assessment of genetic diversity and population structure in wild Ziziphus species from northwest India using SSR marker technique
Source: J Genet Eng Biotechnol. 2023 Jan 13;21:4. doi: 10.1186/s43141-022-00458-6 (PMC9839936; doi:10.1186/s43141-022-00458-6)
Supplement: Supplementary file 1 — Additional file 1: Supplementary Fig. 1 Showing correlation between Genetic and geographic distances of 48 samples used in present study. [file 43141_2022_458_MOESM1_ESM.doc]

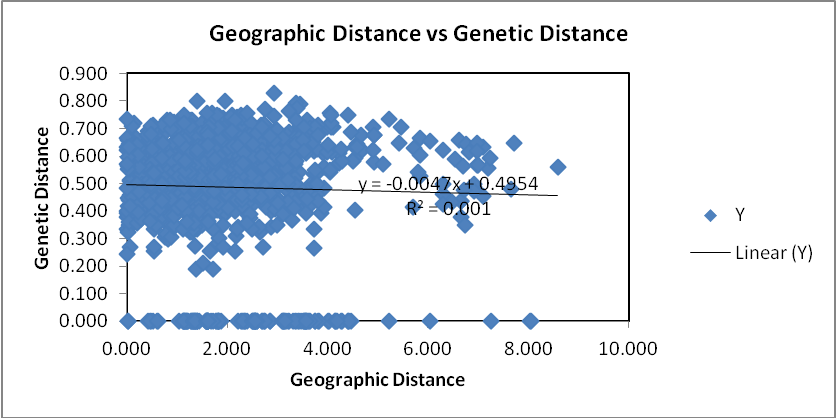


Supplementary Fig.1 Showing correlation between Genetic and geographic distances of 48 samples used in present study.
