# Supplementary material for: Assessment of genetic diversity and population structure in wild Ziziphus species from northwest India using SSR marker technique
Source: J Genet Eng Biotechnol. 2023 Jan 13;21:4. doi: 10.1186/s43141-022-00458-6 (PMC9839936; doi:10.1186/s43141-022-00458-6)
Supplement: Supplementary file 2 — Additional file 2: Supplementary Table 1 Axis wise Eigen values. [file 43141_2022_458_MOESM2_ESM.doc]

**Supplementary Table 1: Axis wise Eigen values**

**Axis Eigen value Inertia%**

Axis Eigen value Inertia%

1 0.02418 20.33

2 0.01071 9.01

3 0.01052 8.84

4 0.00752 6.32

5 0.00673 5.65

**Raw data containing Eigen values**

**Axis 1 Axis 2 Axis 3 Axis 4 Axis 5**

**Coord. Cos² Coord. Cos² Coord. Cos² Coord. Cos² Coord. Cos²**

1 -0.0860 181 -0.0668 109 0.0559 76 0.1031 261 0.0312 24

2 -0.1104 293 -0.0673 109 -0.0269 17 0.0643 99 0.0134 4

3 -0.1557 378 -0.0940 138 -0.0641 64 0.1283 257 -0.0210 7

4 -0.0412 23 -0.0853 99 -0.0212 6 -0.0891 108 0.0118 2

5 -0.1304 490 -0.0115 4 0.0817 192 0.0179 9 -0.0959 265

6 0.1441 285 0.1343 248 -0.1362 255 0.0054 0 0.0294 12

7 0.0145 5 -0.0256 15 0.0106 3 0.1253 356 -0.0492 55

8 -0.1096 213 -0.0769 105 0.0761 103 0.1017 183 -0.0732 95

9 -0.1181 118 0.0594 30 0.1586 212 0.0471 19 -0.2605 573

10 -0.0169 6 0.0028 0 -0.0229 11 -0.0294 19 0.0138 4

11 -0.0820 153 -0.1065 259 -0.0103 2 0.0238 13 0.0588 79

12 -0.1088 265 -0.0553 69 -0.0830 154 0.0229 12 0.0430 41

13 0.0468 40 -0.0041 0 -0.0215 8 0.0711 92 -0.0419 32

14 0.2504 623 -0.0491 24 -0.0053 0 0.0864 74 -0.0300 9

15 0.2535 374 -0.3275 624 -0.0091 0 -0.0255 4 -0.0901 47

16 -0.1677 321 -0.1339 205 -0.0383 17 -0.1118 143 -0.0134 2

17 0.2589 691 -0.0976 98 -0.0687 49 -0.0488 25 -0.1161 139

18 0.2101 537 -0.0473 27 -0.0495 30 -0.1207 177 -0.1210 178

19 0.2590 616 -0.0561 29 0.0703 45 -0.1107 113 -0.1017 95

20 0.2507 706 -0.0402 18 0.0392 17 -0.1033 120 -0.0531 32

21 -0.0580 63 -0.0478 42 0.0386 28 -0.0162 5 0.1898 669

22 0.2073 395 -0.0362 12 0.0194 3 0.0194 3 0.1756 283

23 0.2109 274 0.1864 214 0.0119 1 0.2255 313 0.0201 2

24 0.0832 125 0.0576 60 0.1112 224 0.0190 7 0.0093 2

25 -0.0868 206 -0.0851 198 0.0792 171 0.0069 1 0.0197 11

26 -0.1373 307 -0.1088 193 0.1194 233 -0.0282 13 0.0393 25

27 -0.1270 297 -0.0212 8 0.1154 245 -0.0476 42 0.0632 74

28 -0.1450 414 0.0588 68 0.0723 103 -0.0141 4 0.0409 33

29 -0.1666 475 -0.0014 0 -0.0661 75 0.0082 1 -0.0187 6

30 -0.1256 492 0.0703 154 -0.0005 0 -0.0279 24 -0.0040 0

31 -0.1284 613 -0.0254 24 -0.0194 14 -0.0685 174 0.0569 120

32 -0.1241 356 0.0467 50 0.0934 201 -0.0352 29 0.0463 49

33 0.2031 267 0.1183 91 0.2298 342 -0.0374 9 0.0324 7

34 0.2278 439 0.1369 159 0.0896 68 -0.0706 42 0.0963 78

35 -0.1415 386 0.1097 232 0.0626 76 -0.0679 89 0.0659 84

36 -0.1150 381 0.0597 103 0.0907 237 -0.0378 41 -0.0195 11

37 0.2354 547 0.0201 4 0.1039 106 -0.0556 31 0.0122 1

38 -0.0780 63 0.2494 641 0.0419 18 0.0842 73 -0.0859 76

39 -0.2349 903 0.0008 0 -0.0090 1 0.0464 35 -0.0140 3

40 0.0423 19 0.0962 98 -0.1975 414 0.2062 452 -0.0554 33

41 -0.0967 272 0.0920 246 0.0397 46 -0.0580 98 0.0452 60

42 0.3217 570 0.0264 4 -0.0439 11 0.1090 65 0.1339 99

43 -0.0950 93 0.2089 449 -0.0719 53 -0.0992 101 -0.1078 120

44 -0.1370 438 -0.0386 35 -0.0001 0 -0.1217 345 0.0509 60

45 0.0174 2 0.1492 156 -0.2740 527 -0.1809 230 -0.0636 28

46 -0.0994 106 -0.1478 233 -0.1269 172 0.0986 104 -0.0764 62

47 0.0220 7 -0.0873 105 -0.1069 157 0.0770 82 0.1529 322

48 -0.0357 9 0.0610 27 -0.3380 834 -0.0913 61 0.0604 27
